# Simultaneous Localization and Mapping Algorithm Based on the Asynchronous Fusion of Laser and Vision Sensors

**DOI:** 10.3389/fnbot.2022.866294

**Published:** 2022-05-24

**Authors:** Kexin Xing, Xingsheng Zhang, Yegui Lin, Wenqi Ci, Wei Dong

**Affiliations:** ^1^State Key Laboratory of Robotics and System, Harbin Institute of Technology, Harbin, China; ^2^College of Information Engineering, Zhejiang University of Technology, Hangzhou, China

**Keywords:** laser, vision, SLAM, data fusion, multi-rate

## Abstract

In this paper, a simultaneous localization and mapping algorithm based on the weighted asynchronous fusion of laser and vision sensors is proposed for an assistant robot. When compared to the synchronous fusion algorithm, this method can effectively use the redundant data in the vision sensor and improve the tracking accuracy of the algorithm. At the same time, the attitude estimation of the visual sensor is taken as a prior of the attitude estimation of the laser sensor to reduce the number of iterations and improve the efficiency of the algorithm. Further, according to the running state of the robot, a weighting coefficient based on angle is introduced to improve the confidence of the measurement. Experimental results show that the algorithm is robust and can work in a degraded environment. When compared to the synchronous fusion method, the asynchronous fusion algorithm has a more accurate prior, faster operation speed, higher pose estimation frequency, and more accurate positioning accuracy.

## Introduction

Population aging is a global problem. In 2020, there were 727 million people worldwide aged 65 years and above, and the number of elderly people is expected to more than double over the next 30 years, reaching more than 1.5 billion people by 2050 (Shushpanov et al., 2021). Walking impairments caused by functional decline, cardiovascular disease, or stroke significantly affect the quality of life of several older adults (Owen et al., 2010).

Among medical robots, mobility robots have performed a significant role. For example, the smart wheelchair developed by Karlstad University in Sweden can control movements based on the direction of eye movement and exhibits a navigational ability (Wästlund et al., 2015). The Walking Assistant Robot (Shim et al., 2005) developed by Hyeon-Min et al. of Korea Institute of Technology is equipped with global positioning system sensors and light detection and ranging (LiDAR); therefore, it provides outdoor navigation and obstacle avoidance functions. The autonomous mobile service robot used in nursing homes, which was independently developed by the University of Lincoln in the United Kingdom (Li et al., 2018), uses 3D LiDAR to perceive the surrounding environment and movement of pedestrians, and performs the subsequent movements of the patient based on the perceived data. Sami et al. designed a medical robot (Ramadhan et al., 2021) that is used to perform navigational tasks and basic health tests such as monitoring of the heart rate, oxygen saturation levels, and temperature. In fact the cane type walking-aid robot can be regarded as a typical inverted pendulum system. In order to estimate the disturbance and realize the control stability of the mobile wheeled inverted pendulum systems, a novel sliding mode control based on high-order disturbance observer was developed (Huang et al., 2020). Further a cane type walking-aid robot is proposed (Yan et al., 2021), which follow a human user for the safety and supervision of independent walking during rehabilitation training. The autonomous positioning and navigational capabilities of mobile robots are critical in walking robots, and an accurate map is the basis of these capabilities in walking robots.

In the past few decades, mobile robots based on simultaneous localization and mapping (SLAM) algorithm have been rapidly developed. Laser and vision sensors are the two mainstream sensors used in SLAM applications. Among them, the SLAM algorithms represented by laser sensors primarily include Gmapping (Grisetti et al., 2007), Hector (Kohlbrecher et al., 2011), and cartographer algorithms (Hess et al., 2016). The earliest 3D laser SLAM method is the LiDAR odometry and mapping algorithm proposed by Zhang (Zhang et al., 2016; Zhang and Singh, 2017) of Carnegie Mellon University, which can be used to obtain a laser odometer with higher accuracy and real-time performance and to build an environment map.

With the development of 3D laser sensors, 3D laser sensor schemes have been gradually adopted by automatic driving and outdoor low-speed unmanned vehicles. However, owing to the high cost of 3D laser sensors, 2D lasers are still predominantly used to achieve SLAM in indoor scenes. Karto SLAM (Konoli et al., 2010) is a graph optimisation method in which the map is represented using a graph, and each node represents an anchor point in the trajectory of the robot and a dataset of sensor measurements. Hwang and Song proposed an extended Kalman filter-based SLAM method, which uses the nearest neighbor data association method to obtain data association results, and then uses an extended Kalman filter to estimate the attitude and environment map of a mobile robot (Hwang and Song, 2011). CoreSLAM (Turnage, 2016) uses a Monte Carlo algorithm for scan matching, which outputs low-quality recognizable maps by fusing incoming laser data with the data collected from a laser rangefinder. It was observed from the above study that CoreSLAM performs better on slow robots. Although mapping can be achieved using 2D laser sensors, these sensors have certain drawbacks. Because the 2D laser sensor only has depth information and scans a plane, the algorithm applied in it fails when the depth information returned by the sensor does not change with time during the movement of the robot in the corridor. The camera, another inexpensive sensor, has obvious advantages in this case. On the one hand, it can obtain significant corner or edge information in stereo space. On the other hand, cameras are widely used because of their higher sampling rates. MonoSLAM (Davison et al., 2007) is the first SLAM system for the real-time recovery of monocular vision; it uses an extended Kalman filter as a back-end to track sparse features in the front-end, thus updating the current state of the camera and all feature points of state variables. Klein and Murray (2007) proposed a parallel tracking and mapping (PTAM) algorithm that operates in real time, where camera pose tracking and feature point map creation are performed in parallel. Systems designed with this algorithm can generate detailed maps containing thousands of road signs that can be tracked at faster frame rates with accuracy and robustness when compared to state-of-the-art model-based systems. De Croce et al. (2019) proposed distributed stereo PTAM (DS-PTAM), a distributed architecture for stereo PTAM, which is a stereo SLAM system, in which the input information is acquired by two cameras, and the tracking and mapping processes are performed in a distributed system. The DS-PTAM system allows positioning and mapping modules to be run on remote base stations, thereby reducing the load on the vehicle processor. However, the camera also has its own shortcomings, and it is easy to lose features and cause greater drift when rotating over a large range. In this case, the laser exhibits superior properties.

In recent years, because each sensor has its own limitations, researchers have developed many SLAM mapping algorithms based on multi-sensor fusion. Shamwell et al. (2020) adopted an unsupervised neural network approach to fuse RGB-depth (RGB-D) images with absolute trajectory estimation from inertial measurements. The network learns to integrate the measurements of an inertial measurement unit (IMU) and generates hypothetical trajectories, which are corrected online based on a Jacobian matrix of the scaled image projection error relative to the pixel coordinate space grid. Westfechtel et al. (2019) used the synchronous LiDAR odometry and mapping algorithm (Zhang and Singh, 2014) to synchronize the LiDAR scanning and camera data, and used a neural network algorithm to project 3D laser data onto 2D image data to collect semantic information of the image and constructed 3D RGB-SLAM. Oriented features from accelerated segment test and rotated binary robust independent elementary features (ORB)-SLAM (Mur-Artal et al., 2015) computations revolve around fast orientation and rotational brevity, including an ORB dictionary for visual velocimetry and loop detection; moreover, it innovatively utilizes three threads to achieve SLAM. In another study (Zhang and Singh, 2018), the LiDAR visual-inertial odometry algorithm was proposed, which fused multi-line LiDAR, camera, and IMU sensors. This algorithm used the constraints of camera images and IMU measurements to achieve high-frequency attitude output; then, it used feature matching among laser point clouds to achieve low-frequency attitude output and output mapping to achieve a higher-frequency attitude output; finally, a 3D point cloud map was constructed. Shan et al. (2020) proposed LiDAR inertial odometry *via* smoothing and mapping, a tightly coupled laser radar inertial odometer framework that enables highly accurate, real-time mobile robot trajectory estimation and map construction through smoothing and mapping. LiDAR inertial odometry *via* smoothing and mapping can effectively improve the real-time performance of a system in the local range. In another study (Qiu et al., 2008), a modular 3D tracking system for six-degrees-of-freedom pose estimation of dynamic objects with metric scale recovery capability using monocular visual-inertial sensing was proposed. The tracking system could deal with a general rigid object based on motion correlation analysis without scale prior processing of the tracked object. Liu and Shen (2017) proposed a spline-based monocular visual-inertial navigation system-based altitude estimator initialization method. This method can explicitly reduce the number of parameter estimates required to achieve earlier convergence. Qin et al. (2018) proposed a monocular vision-inertial SLAM system that can reposition the camera and obtain the absolute pose of the camera from the existing map. Then, a four-degrees-of-freedom pose graph is optimized to correct the drift and achieve global consistency. However, there are very few algorithms for the fusion of vision and lasers. The identification of a method that can fuse the two sensors with strong complementary advantages to achieve efficient and accurate mapping and navigation is important. Zuo et al. (2019) used EKF to fuse the information of laser, IMU and visual odometer, but the different speed and characteristics for different sensors are not considered. Mu (2020) used Kalman filter (UKF) to designed the related strategy of the 2D LiDAR point cloud and RGB-D camera point cloud. the experiment shows that the multi-sensor SLAM framework designed has a good effect. Geneva et al. (2018) designed a factor graph-based optimization framework to realize the asynchronous fusion of laser and vision. The proposed sensor-fusion system got an improved performance on a real-world experimental dataset. But these methods do not consider the difference degree of confidence between laser and vision in different scenes. For example, the laser and the vision have different sensitiveness during turning and straight-line walking. Our work will also consider the confidence difference caused by characteristics of sensors expect the asynchronous fusion. It not only promises the sensors can operate at different sampling rates, but also effectively avoids performance loss caused by a specific scene.

The primary contributions of this paper are as follows. Based on 2D laser and binocular cameras, a weighted asynchronous fusion algorithm is proposed. The algorithm considers the acquisition frequency characteristics of different sensors and ensures that different sensors have different confidence levels in different operating states. Finally, the effectiveness of the algorithm is proven based on the results of simulations and experiments. The experiments showed that the proposed algorithm has good accuracy and robustness.

The rest of this paper is organized as follows. Section Algorithm presents the modeling of asynchronous multi-rate multi-sensor system. Section Experimental Results presents the detailed simulation and experimental results. Section Conclusion concludes the study.

## Algorithm

### Modeling of Asynchronous Multi-Rate Multi-Sensor System

For the pose increment obtained based on the feedback from the vision sensor, according to the pinhole imaging principle of the camera and the rigid body transformation formula, it is assumed that the homogeneous coordinate of a space point P in the world coordinate system is $P={\left(X_{W},Y_{W},Z_{W},1\right)}^{T}$. Then, its coordinates in the pixel coordinate system of the camera are obtained as follows:

(1) $$\begin{array}{l} s\left[\begin{array}{c} u\\ v\\ 1 \end{array}\right]=K\left[\begin{array}{cc} R & t\\ 0 & 1 \end{array}\right]\left[\begin{array}{c} X_{W}\\ Y_{W}\\ Z_{W}\\ 1 \end{array}\right] \end{array}$$

where (u, v) is the coordinate of point P with respect to the pixel coordinate system, s is the depth of point P with respect to the camera coordinate system, K is the internal parameter matrix of the camera, R is the rotation matrix, and t is the translation matrix.

The feature points of the image in the current frame can be obtained through the ORB feature extraction algorithm (Turnage, 2016), and the descriptor of the feature points in the current frame obtained by the binary robust independent elementary feature descriptor is matched with the feature points in the image at the previous moment; then, the corresponding matching relationship is established. The matching point at the previous moment is projected into the current frame of the image through the internal parameter matrix of the camera and the initial pose. Because the pose of the camera usually exhibits a certain deviation, there is usually a certain deviation between the projected point and the matching point on the current map. Assuming that the coordinates of the feature point P in the current frame image are $P={\left(u_{1},v_{1}\right)}^{T}$, the coordinates of the matching point in the current frame corresponding to the matching point in the previous frame is $P^{\prime }={\left(u_{2},v_{2}\right)}^{T}$. Because the deviation of the camera pose usually causes the coordinates of these two points to be different, the error function is defined as follows:

(2) $$\begin{array}{l} e=\left[\begin{array}{c} u\\ v \end{array}\right]-\frac{1}{s}K\left[\begin{array}{cc} R & t\\ 0 & 1 \end{array}\right]P, \end{array}$$

where e is the error, (u, v) is the pixel coordinate of the feature point in the current frame, and P is the matching point corresponding to the feature point in the current frame. Equation (2) can be rewritten into the Lie algebra form as follows:

(3) $$\begin{array}{l} e=\left[\begin{array}{c} u\\ v \end{array}\right]-\frac{1}{s}Kexp\left(\delta \right)P, \end{array}$$

where exp(δ) is the Lie algebra expression of the camera coordinate system and e is the projection error between the space point P projected onto the current image and the matching point. There are usually several matching points in a frame of the image, and according to all the matching points, the projection error function can be obtained as follows:

(4) $$\begin{array}{l} \delta^{*}=\overset{N}{\underset{i=1}{\sum }}|u_{i}-\frac{1}{s_{i}}Kexp(\delta )P_{i}{|}^{2}, \end{array}$$

where *u*_*i*_ represents the i-th feature point in the current frame, *P*_*i*_ represents the world coordinate of its corresponding matching point, *s*_*i*_ represents the depth of the point, K represents the internal parameter matrix of the camera, exp(δ) represents the Lie algebra expression of the camera coordinate system.

Using the Gauss–Newton or Levenberg–Marquardt method to solve the optimisation equation, the pose estimate is obtained as

(5) $$\begin{array}{l} z_{1}\left(k_{1}\right)=\left[\begin{array}{cc} R & t\\ 0 & 1 \end{array}\right]+v_{1}\left(k_{1}\right), \end{array}$$

where *z*_1_(*k*_1_) is the optimal estimation of the visual sensor at time *k*_1_, R is the rotation matrix, and t is the translation matrix. *v*_1_(*k*_1_) is the estimated noise of the camera and is assumed to follow a Gaussian distribution.

Let us assume that the laser sensor data obtained at time *k* is $S_{i}{\left(x_{i},y_{i}\right)}^{k}$; let *S*_*i*_(δ_2_) be the laser sensor data corresponding to the coordinates δ_2_. δ_2_ is the coordinate of the robot in the world coordinate system at the last cycle. *M*(*S*_*i*_(δ_2_)) is the occupancy probability of point *S*_*i*_(δ_2_) and ∇*M*(*S*_*i*_(δ_2_)) is the derivative of *M*(*S*_*i*_(δ_2_)). The values of *M*(*S*_*i*_(δ_2_)) and ∇*M*(*S*_*i*_(δ_2_)) can be obtained by performing bilinear interpolation on the world coordinate *S*_*i*_(δ_2_). Then, the pose increment of the robot from the previous moment to the current moment can be given by the following formula:

(6) $$\begin{array}{l} \delta_{2}=H^{-1}\overset{n}{\underset{i=1}{\sum }}{\left[\nabla M\left(S_{i}\left(\delta_{2}\right)\right)\frac{\partial S_{i}\left(\delta_{2}\right)}{\partial \delta_{2}}\right]}^{T}\left(1-M\left(S_{i}\left(\delta_{2}\right)\right)\right). \end{array}$$

According to the pose increment, the coordinates of the robot under the pose estimation performed by the laser sensor can be obtained as

(7) $$\begin{array}{l} z_{2}\left(k_{2}\right)=z_{2}\left(k_{2}-1\right)+\delta_{2}+v_{2}\left(k_{2}\right), \end{array}$$

where *z*_2_(*k*_2_) is the optimal pose estimation of the laser sensor at time *k*_2_ *and z*_2_(*k*_2_ − 1) is the optimal pose estimation at the previous moment, *v*_2_(*k*_2_) is the observation noise, which is assumed to obey the Gaussian distribution; therefore, it satisfies the following relationship:

(8) $$\begin{array}{l} \{\begin{array}{c} E\left[v_{i}\left(k_{i}\right)\right]=0\\ E\left[v_{i}\left(k_{i}\right){v_{j}}^{T}\left(k_{j}\right)\right]=R_{i}\left(k_{i}\right)\delta_{ij}\delta_{k_{i}k_{j}} \end{array} \end{array}$$

The traditional fusion method involves the fusion of the two sensors after obtaining the optimal estimate of the two sensors; however, the rates of the two sensors are usually not the same. For example, the frequency of the simulated camera used in the subsequently described experiments was approximately 15 Hz, and the frequency of the simulated radar was approximately 6 Hz. If timestamp alignment is used, a large amount of data obtained from the faster sensor will not be utilized effectively. Therefore, this study adopts an asynchronous fusion method to fuse data from two sensors.

First, the sensor data were divided into m sampling intervals according to the sampling frequency of the faster sensor. There are two situations in each sampling interval:

1. Only the pose estimation of the camera is included
2. The pose estimation from both the laser and vision sensors are included.

By combining the Kalman filter and multi-sensor asynchronous fusion method, the improved pose estimation algorithm based on asynchronous fusion of laser and vision data is obtained as follows:

(9) $$\begin{array}{l} \{\begin{array}{c} {\hat{x}}_{i}\left(k|k\right)={\hat{x}}_{i-1}\left(k|k\right)+K_{i}\left(k\right)\left[z_{i}\left(k_{i}\right)-{\hat{x}}_{i-1}\left(k|k\right)\right]\\ P_{i}\left(k|k\right)=P_{i-1}\left(k|k\right)-K_{i}\left(k\right)P_{i-1}\left(k|k\right)\\ K_{i}\left(k\right)=P_{i-1}\left(k|k\right){\left[P_{i-1}\left(k|k\right)+R_{i}\left(k\right)\right]}^{-1} \end{array} \end{array}$$

where ${\hat{x}}_{0}\left(k|k\right)$ is the predicted pose obtained from the optimal estimation of the robot pose at the previous moment, *P*_0_(*k*|*k*) is the covariance matrix of the predicted pose, *z*_*i*_(*k*_*i*_) is the optimal pose estimation from the laser or vision sensor at time k.

When there is only camera data in the sampling interval, then I = 1, and Equation (9) is rewritten as follows:

(10) $$\begin{array}{l} \{\begin{array}{c} {\hat{x}}_{1}\left(k|k\right)={\hat{x}}_{0}\left(k|k\right)+K_{1}\left(k\right)\left[z_{1}\left(k_{1}\right)-{\hat{x}}_{0}\left(k|k\right)\right]\\ P_{1}\left(k|k\right)=P_{0}\left(k|k\right)-K_{1}\left(k\right)P_{0}\left(k|k\right)\\ K_{1}\left(k\right)=P_{0}\left(k|k\right){\left[P_{0}\left(k|k\right)+R_{1}\left(k\right)\right]}^{-1} \end{array} \end{array}$$

At this time, the estimation model is transformed into a Kalman filter that performs pose estimation for the data from a single sensor at a faster sampling rate.

When both the camera data and the pose estimation result of the laser sensor exist in the sampling interval, Equation (9) is modified as follows.

(11) $$\begin{array}{l} \{\begin{array}{c} {\hat{x}}_{1}\left(k|k\right)={\hat{x}}_{0}\left(k|k\right)+K_{1}\left(k\right)\left[z_{1}\left(k_{1}\right)-{\hat{x}}_{0}\left(k|k\right)\right]\\ P_{1}\left(k|k\right)=P_{0}\left(k|k\right)-K_{1}\left(k\right)P_{0}\left(k|k\right)\\ K_{1}\left(k\right)=P_{0}\left(k|k\right){\left[P_{0}\left(k|k\right)+R_{1}\left(k\right)\right]}^{-1} \end{array} \end{array}$$

(12) $$\begin{array}{l} \{\begin{array}{c} {\hat{x}}_{2}\left(k|k\right)={\hat{x}}_{1}\left(k|k\right)+K_{2}\left(k\right)\left[z_{2}\left(k_{2}\right)-{\hat{x}}_{1}\left(k|k\right)\right]\\ P_{2}\left(k|k\right)=P_{1}\left(k|k\right)-K_{2}\left(k\right)P_{1}\left(k|k\right)\\ K_{2}\left(k\right)=P_{1}\left(k|k\right){\left[P_{1}\left(k|k\right)+R_{2}\left(k\right)\right]}^{-1} \end{array} \end{array}$$

**Figure 1:**
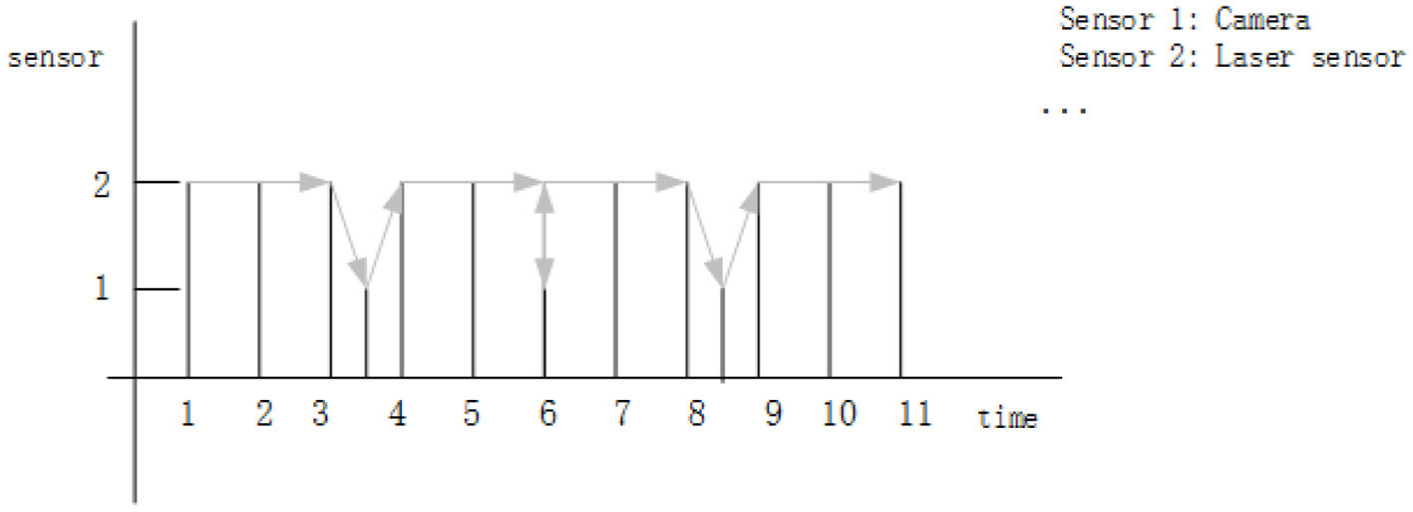
Schematic diagram of sensor fusion.

At this time, the estimation model is transformed into the Kalman filter algorithm that fuses and performs pose estimation of the data from the laser and vision sensors at a slower sampling rate. The algorithm uses the optimal estimation of the previous moment as the prediction quantity. First, the pose estimated by sensor 1 (camera) at the current moment is fused; then, the pose results estimated by sensor 2 (laser sensor) are fused; finally, the sensor estimation value after two fusions is used as the final pose estimation result. The schematic diagram of sensor fusion is shown in the Figure 1. The arrow indicates the update order of the observation value when obtaining the optimal estimation of the state.

### Modeling of Weighted Asynchronous Multi-Rate Multi-Sensor System

In the previous fusion algorithms, the characteristics of different sensors were usually not considered. For example, in the process of pose estimation using only laser data, when the robot enters a scene with inconspicuous structural features such as a corridor, the sensor data obtained by the robot during the movement along the corridor may not change with time and the movement of the robot. At this time, the accuracy of the pose estimation obtained by the laser sensor is significantly reduced. If the pose is still fused under normal conditions, it will lead to a large estimation error. Similarly, when the vision is rotated, if the feature points are not sufficiently obvious or the number of feature points is insufficient, then the pose estimation from the vision is prone to drift.

To effectively utilize the different characteristics of each sensor, the concept of confidence is introduced according to this characteristic of the sensor. When a robot walks in the corridor, the influence of the deviation of the laser sensor data is reduced as much as possible, and the pose drift problem caused by the camera drift when the robot is turned is reduced to the maximum extent possible.

By using the confidence w to represent the confidence in the pose estimates of the two sensors, Equation (9) can be transformed as follows:

(13) $$\begin{array}{l} \{\begin{array}{c} {\hat{x}}_{i}\left(k|k\right)={\hat{x}}_{i-1}\left(k|k\right)+w_{i}K_{i}\left(k\right)\left[z_{i}\left(k_{i}\right)-{\hat{x}}_{i-1}\left(k|k\right)\right]\\ P_{i}\left(k|k\right)=P_{i-1}\left(k|k\right)-K_{i}\left(k\right)P_{i-1}\left(k|k\right)\\ K_{i}\left(k\right)=P_{i-1}\left(k|k\right){\left[P_{i-1}\left(k|k\right)+R_{i}\left(k\right)\right]}^{-1} \end{array}, \end{array}$$

where *w*_*i*_ represents the pose estimation confidence of the i-th sensor.

The characteristics of the sensor are usually related to the motion of the robot. When the robot walks in a straight line, the laser sensor often experiences positioning problems, and the camera often drifts when turning. Therefore, *w*_*i*_ should be related to the motion state of the robot. When the robot moves in a straight line, the algorithm increases the confidence of the data from the camera, and when the robot rotates, the algorithm increases the confidence of the data from the laser.

In each fusion, the algorithm determines whether the robot rotates during the current motion according to the latest pose estimation of the laser sensor. This is obtained using Equation (6) and is denoted as α_2_.

By substituting the angle change into w, we obtain:

(14) $$\begin{array}{l} \{\begin{array}{c} w_{1}=\alpha_{2}\\ w_{2}=1-\alpha_{2} \end{array} \end{array}$$

where α_2_ is the estimated rotation angle of the robot from the previous moment to the current moment. According to the current operation effect of the robot, the rotation angle of the robot does not exceed 0.25 rad in each sampling period. Therefore, Equation (14) can be transformed as follows.

(15) $$\begin{array}{l} \{\begin{array}{c} w_{1}=4\alpha_{4}\\ w_{2}=1-4\alpha_{4} \end{array} \end{array}$$

By substituting (15) into (13), we obtain:

(16) $$\begin{array}{l} \{\begin{array}{c} {\hat{x}}_{1}\left(k|k\right)={\hat{x}}_{0}\left(k|k\right)+4\alpha_{1}K_{1}\left(k\right)\left[z_{1}\left(k_{1}\right)-{\hat{x}}_{0}\left(k|k\right)\right]\\ P_{1}\left(k|k\right)=P_{0}\left(k|k\right)-K_{1}\left(k\right)P_{0}\left(k|k\right)\\ K_{1}\left(k\right)=P_{0}\left(k|k\right){\left[P_{0}\left(k|k\right)+R_{1}\left(k\right)\right]}^{-1} \end{array}, \end{array}$$

(17) $$\begin{array}{l} \{\begin{array}{c} {\hat{x}}_{2}\left(k|k\right)={\hat{x}}_{1}\left(k|k\right)+{\left(1-4\alpha_{1}\right)K}_{2}\left(k\right)\left[z_{2}\left(k_{2}\right)-{\hat{x}}_{1}\left(k|k\right)\right]\\ P_{2}\left(k|k\right)=P_{1}\left(k|k\right)-K_{2}\left(k\right)P_{1}\left(k|k\right)\\ K_{2}\left(k\right)=P_{1}\left(k|k\right){\left[P_{1}\left(k|k\right)+R_{2}\left(k\right)\right]}^{-1} \end{array} \end{array}$$

When compared to the synchronous fusion method, this method first improves the data utilization rate of the visual sensor, and then utilizes the varied characteristics of different sensors; consequently, the algorithm has a better utilization rate for different sensors under different conditions, and results in more accurate system tracking effect. The algorithm flow is as Algorithm 1.

**Algorithm 1:** Weighted asynchronous fusion SLAM algorithm.

**Input** 1. Determine the situation in the sampling space 2. If there is only camera data in the sampling interval, i=1, then the estimation model is transformed into a Kalman filter for pose estimation from a single sensor data at the camera sampling rate [Eq.(10)], otherwise the sampling interval has pose estimates from both the laser sensor and the vision sensor, then 3. (1) Take the frequency of the laser sensor as the sampling rate and as the pre-measure
(2) Fusion of the pose estimated by the camera at the current moment
(3) Refuse the pose result estimated by the laser sensor, and finally use the sensor estimation value after two fusions as the final pose estimation result [Eq.(11) – (12)] 4. According to the operating state of the robot, an angle-based weighting factor is introduced [Eq. (13)]. If the robot moves in a straight line, then increase confidence in vision [Eq. (16)]. Otherwise when the robot appears to rotate, then increase confidence in lasers [Eq. (17)] 5. Let k=k+1, return 1 **Output**

## Experimental Results

### Gazebo Simulation Environment Experiment

To verify the effectiveness of the algorithm, experiments were conducted in a gazebo simulation environment and a real environment. In this section four kind of SLAM algorithms are applied to a robot model equipped with vision and laser sensors. The results are compared to find the best accuracy method.

**Figure 2:**
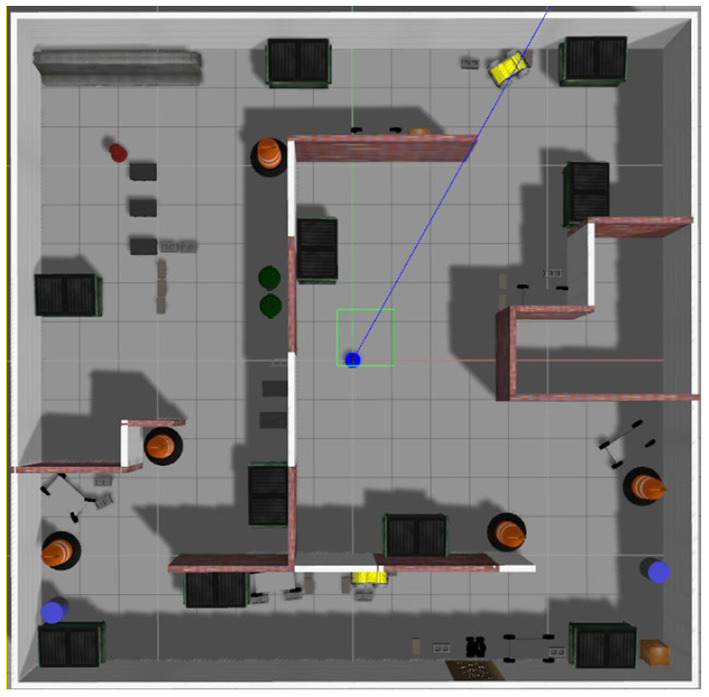
Gazebo simulation environment.

Figure 2 shows a randomly built gazebo simulation environment, where the simulation environment is a 16 m × 16 m loop-shaped space. Most of the environment in this space is a normal mapping environment; however, a long corridor at the bottom is a degraded environment. In this corridor, the laser obtains less feature information. The blue point in Figure 2 represents the robot model, in which a simulated LiDAR and a binocular camera are installed. The LiDAR calls the libgazebo_ros_laser.so plugin with a maximum scanning distance of 6 m and maximum scanning angle of 4 rad (~230°). The simulated noise is a Gaussian noise with a mean of 0 and variance of 0.01. The camera model calls the libgazebo_ros_multicamera.so plugin with a horizontal field of view of 1.39 rad, square image size with dimensions of 800 × 800; moreover, the simulated noise is a Gaussian noise with mean of 0 and variance of 0.007.

**Figure 3:**
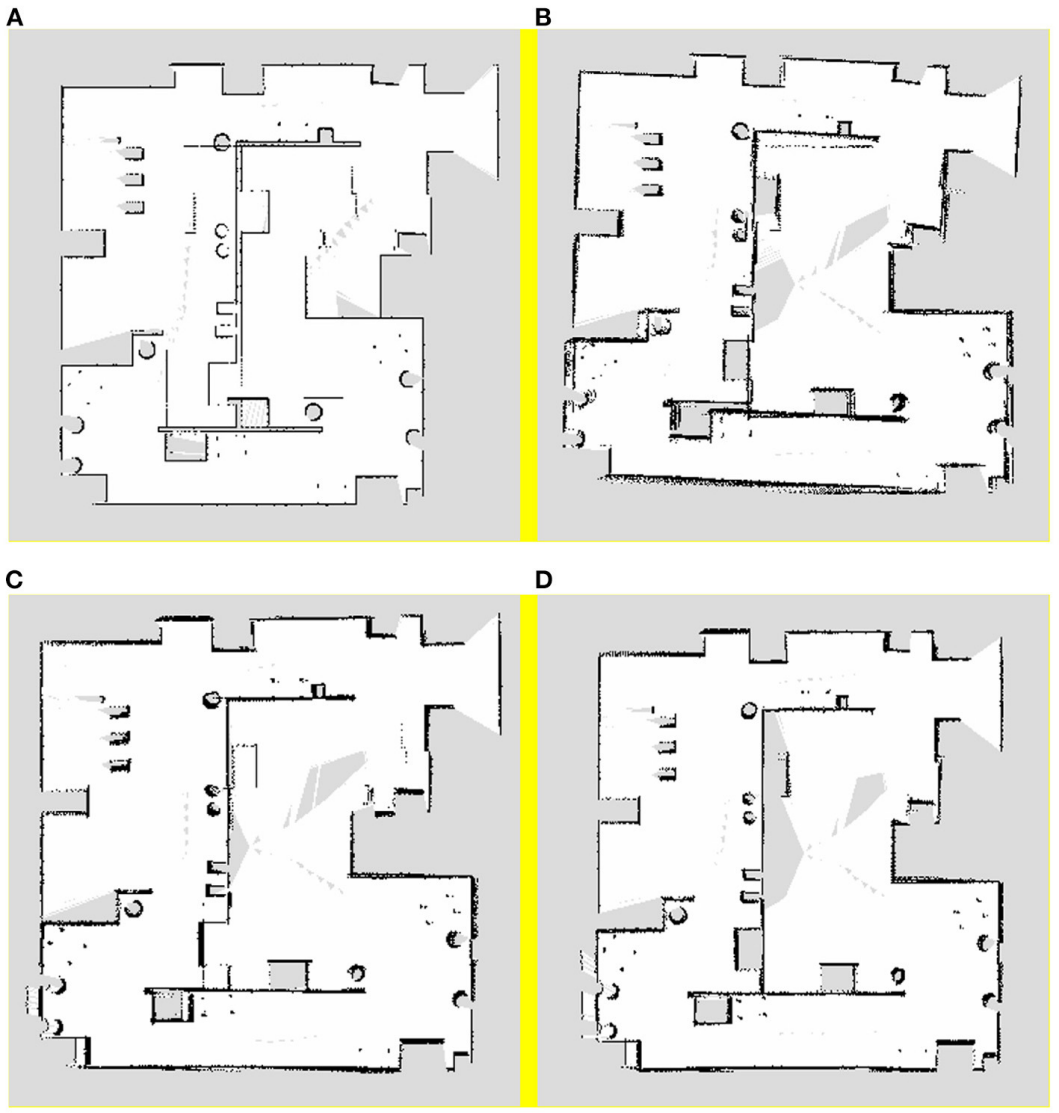
Mapping results of **(A)** pure laser algorithm, **(B)** visual pose estimation algorithm, **(C)** synchronous Kalman filter algorithm, and **(D)** asynchronous Kalman filtering and weighting algorithm.

A traditional pure laser algorithm, visual pose estimation algorithm, synchronous Kalman filter algorithm, and asynchronous Kalman filtering and weighting algorithm, which is the proposed algorithm, were used for 2D SLAM mapping in the simulation map. The mapping results of the aforementioned algorithms are shown in Figures 3A–D, respectively.

It can be seen from Figure 3A that there is a large error in the simple laser algorithm mapping, which is primarily caused by the degraded environment in the lower part of the map. In this environment, because the data returned by the robot from the laser sensor do not change with time during the movement, the robot has a large error in its own pose estimation, which leads to the mapping error of the entire map.

When compared to the mapping result of the laser sensor, it can be observed from Figure 3B that the visual pose estimation is more stable and the map reflects the real scene. However, although the visual pose estimation has a certain accuracy in the degraded environment, owing to the mismatching problem that occurs during the process of rotation in the visual algorithm, the map boundary is more blurred, resulting in the problem of multiple boundaries.

From Figure 3C, it can be observed that the result of the Kalman filtering algorithm is better than the mapping results shown in Figures 3A,B. When compared to the mapping results obtained by the pure laser algorithm, the pose estimation of this algorithm in the degraded environment is more accurate; some optimisations have been incorporated; consequently, the image boundaries of this method are relatively more stable. However, this method still exhibits certain limitations. First, there is still a certain error in the degraded environment. Although the boundary has been improved, there is a problem with multiple boundaries.

As shown from Figure 3D, the asynchronous Kalman filtering and weighting algorithm has best tracking accuracy than other algorithms. Moreover, it has better robustness in the case of degraded environment or robot rotation.

**Figure 4:**
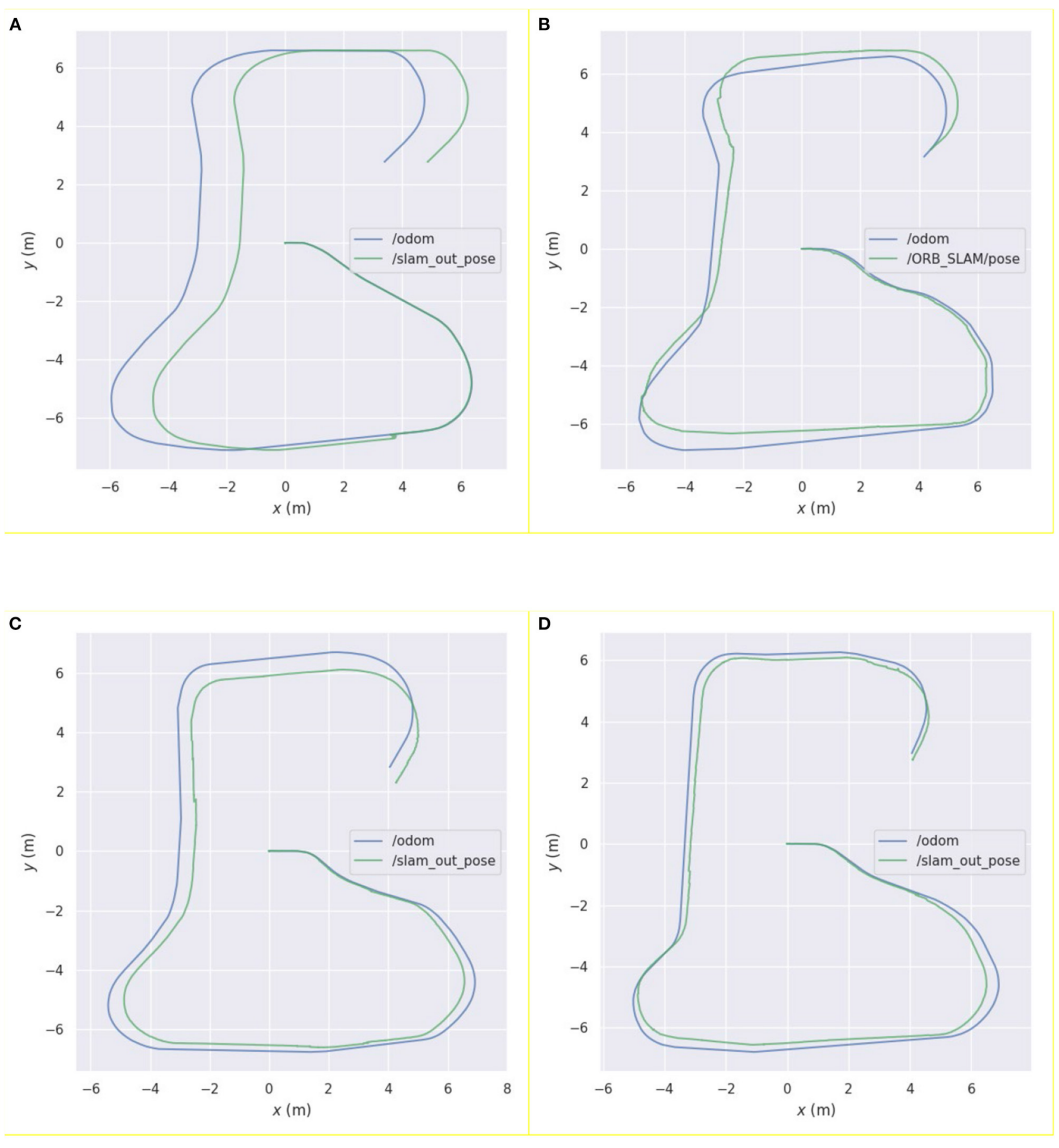
Comparison between the pose curve of the real robot and the pose curves estimated by the following algorithms: **(A)** Hector laser simultaneous localization and mapping (SLAM) algorithm; **(B)** ORB_SLAM algorithm; **(C)** Kalman filter algorithm; **(D)** asynchronous weighted Kalman filter algorithm.

Further, this study also compared the pose estimation performance of the above four algorithms in a specific environment. The results are shown in Figure 4. Figures 4A,B show the running trajectory of the pure laser and pure vision algorithms and the corresponding real motion trajectories of the robot, respectively. Figure 4C shows a comparison of the fusion pose estimation obtained by the Kalman filter algorithm using the visual and laser pose estimations with the motion trajectory of the real robot. Figure 4D shows a comparison between the fusion pose estimation obtained by the weighted asynchronous fusion Kalman filter algorithm and the motion trajectory of the real robot using the visual and laser pose estimations.

From Figure 4A, it can be observed that, before the robot moves to the degraded environment, the running trajectory of the pure laser algorithm in this environment coincides significantly with the actual trajectory, which reflects the movement trajectory of the robot. When the robot enters the degraded environment, the algorithm demonstrates obvious errors in its own posture judgement, indicating that the robot has a significant problem in the positioning of the area; after the robot leaves the area, the error is maintained within a certain range until the end of the movement.

In Figure 4B, it can be noted that the error in pose estimation is more obvious than that in the laser algorithm. The robot exhibits certain deviations in the movement process, especially when the robot rotates, and the error changes more obviously. Before the loop closure, the error gradually increases with the increase in the moving distance of the robot.

In Figure 4C, it can be observed that the visual error has been improved using laser pose estimation; however, it can be observed from the figure that its running error in the degraded environment is still obvious, and the wrong pose estimation of the laser sensor affects the entire algorithmic pose estimation. Modifying the noise term reduces this error but increases its error in other scenarios.

When compared to the other estimation curves, it can be observed from Figure 4D that the pose estimation curve of this algorithm has a higher degree of fit with the actual motion curve of the robot, and the robot exhibits a higher positioning accuracy.

**Figure 5:**
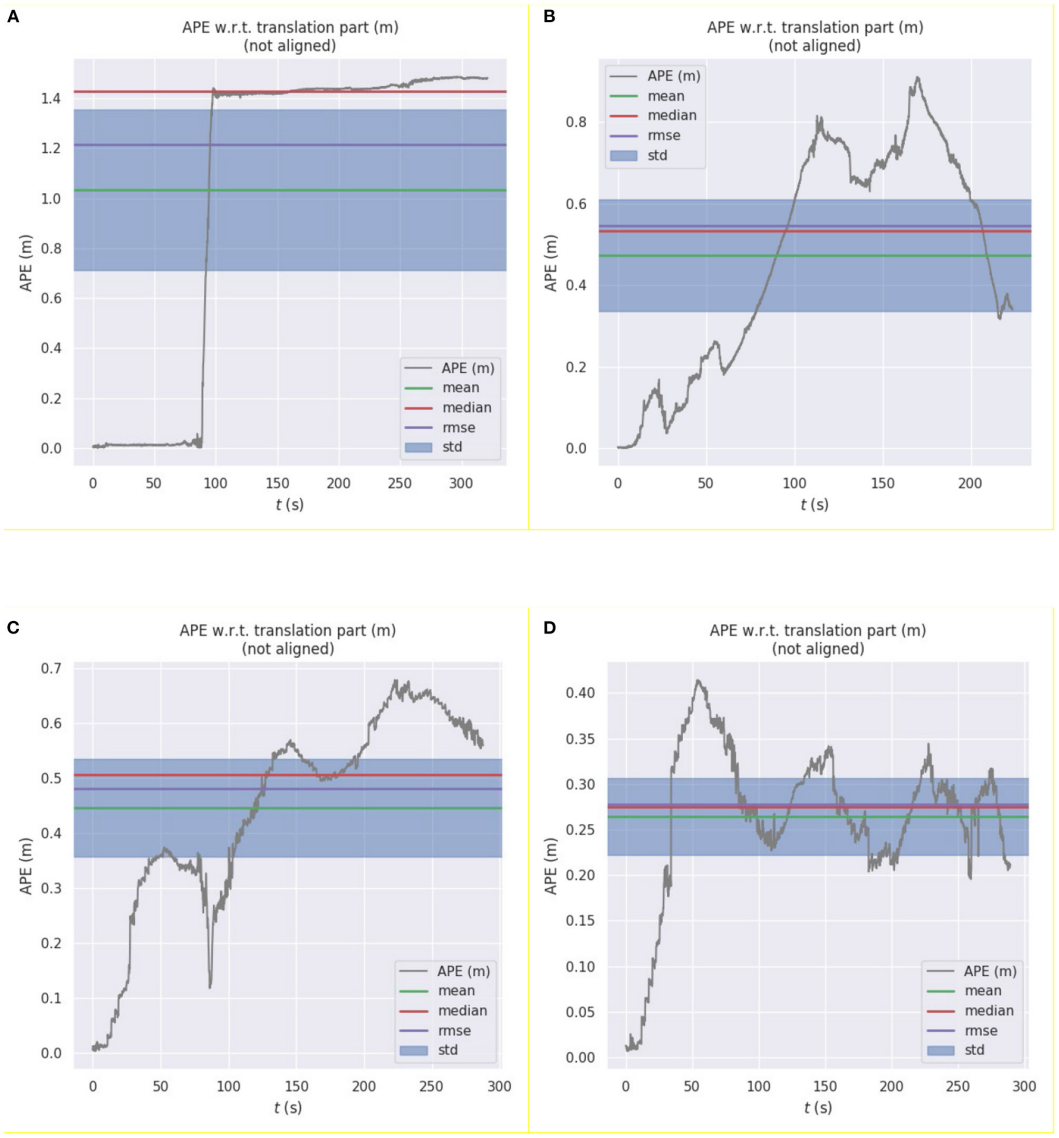
Error curves between the pose of the real robot pose and the pose estimated by the following algorithms: **(A)** Hector laser SLAM algorithm; **(B)** ORB_SLAM algorithm; **(C)** Kalman filter algorithm; **(D)** asynchronous weighted Kalman filter algorithm.

To compare the error between the curves estimated by the algorithms and the real trajectory in Figures 4A–D, we used the evo_ape function in the evo tool to calculate the deviation between the two curves and plotted graphs, as shown in Figure 5.

From the error curves, we can observe the pose estimation effects of the four algorithms. Among them, the error of the pure laser SLAM algorithm increases sharply in the degraded environment, and the error of the pure visual SLAM algorithm gradually accumulates during the movement process. The pose estimation results based on Kalman filtering are optimized; however, the error is still large. When compared to the ordinary Kalman filter algorithm, the pose estimation result of the weighted Kalman filter algorithm is more effective for the pose estimation results of these two sensors, and the overall error is the smallest.

The statistical results of the pose estimation errors of the four algorithms are listed in Table 1.

**Table 1:** Laser SLAM, Vision SLAM, Kalman filtering algorithm, and Weighted Kalman filtering algorithm

	**Laser SLAM**	**Vision SLAM**	**Kalman**	**Weighted Kalman**
			**filtering**	**filtering**
			**algorithm**	**algorithm**
Maximum error	1.485675	0.911249	0.679231	0.414312
Mean error	1.032539	0.472870	0.447091	0.264413
Median error	1.428655	0.532956	0.507386	0.274588

In summary, in terms of the accuracy of the above four algorithms, the weighted asynchronous fusion algorithm has a good positioning effect in both normal and degraded environments, and the overall accuracy of the algorithm is the best among the four algorithms.

In the visual and laser pose estimations, the algorithms use nonlinear optimisation to obtain the optimal pose estimation. The number of iterations and results of the nonlinear optimisation are closely related to the initial value. A good initial value can accelerate the convergence speed and prevent it from falling into a local minimum. In the traditional laser SLAM algorithm, because only one laser sensor is used to estimate the pose, more pose information cannot be provided. Therefore, in the laser SLAM algorithm, each pose update is usually the optimal pose estimation of the previous moment, which the algorithm considers as the initial value of the pose of the robot at the current moment. The robot is in a continuous motion process; therefore, the initial value is usually far from the real value, and it requires several iterations to reach the real value.

In the proposed algorithm, the pose estimation characteristics of asynchronous sensors are completely considered. When the frequency of the sensor is different, the latest pose of the sensor with a higher frequency is used as the pose estimation prior of the sensor with a lower frequency. Therefore, the algorithm has a more accurate prior and the operation speed of the algorithm is improved by reducing the number of iterations of the algorithm.

To compare the computing speed of the two algorithms, the time required for each operation over a period of time and the number of iterations of the algorithm were extracted, as listed in Table 2.

**Table 2:** Algorithm operation time and iteration times.

**Step size**	**0.01**			**0.008**		
	**Time consumption (ms)**	**Iteration times a**	**Iteration times b**	**Time consumption (ms)**	**Iteration times a**	**Iteration times b**
Kalman filtering	13.4	5.2	3.8	17.9	5.5	6.8
Asynchronous Kalman filtering	11.3	4.6	3.8	12.8	5.3	4.4

The search step listed in Table 2 was obtained by an iterative calculation in the nonlinear optimisation process. Because nonlinear optimisation is a process of continuous iterations and infinite approximations to the optimal value, the algorithm sets a threshold. When the number of iterations or the step size of an iteration is less than a certain fixed value, the pose estimation of the algorithm is considered to be sufficiently accurate, and the iterative process is stopped at this time. This paper discusses the time consumption when using the Kalman filtering and asynchronous Kalman filtering algorithms under different thresholds. When the threshold is 0.01, the average Kalman filter algorithm takes 13.4 ms on average, whereas the asynchronous Kalman filter algorithm requires only 11.3 ms on average in the case of a better prior. When the threshold is reduced to 0.008, the period in the ordinary Kalman filtering algorithm increases to 17.9 ms, whereas the period in asynchronous Kalman filtering algorithm increases to 12.8 ms. It can be observed that the operation speed of the algorithm has a definite improvement in the case of better prior.

The number of iterations in Table 2 represents the average number of iterations required till the algorithm step size reaches the threshold. In the laser SLAM algorithm, because the multi-resolution map is used for pose estimation, the algorithm first performs a nonlinear optimisation at low resolution (corresponding to the number of iterations “a” in Table 2), and then improves the resolution when the step length reaches the threshold (corresponding to the number of iterations “b” in Table 2). The algorithm stops iterating when the optimisation step reaches the threshold under the high-resolution map. From the results listed in Table 2, it can be observed that the iterative period of the asynchronous Kalman filtering algorithm is less than that of the ordinary Kalman filtering algorithm under both step-size thresholds. This shows that sufficiently accurate priori conditions can reduce the number of iterations of the algorithm, thereby improving the speed of the algorithm.

### Comparative Experiment in a Real Environment

In addition to the experiment in the simulation environment, comparative experiments between the two algorithms were performed in a real environment.

**Figure 6:**
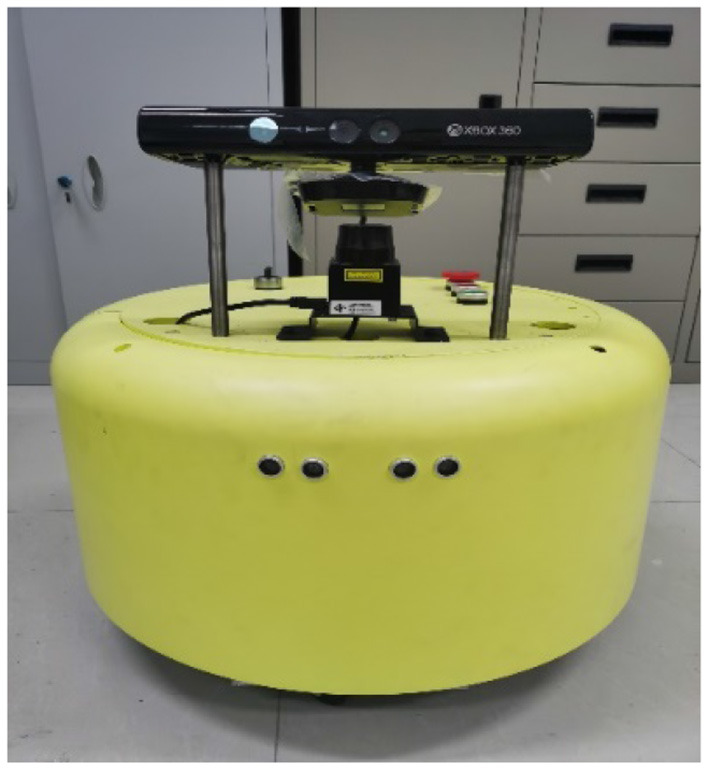
Robot in real scene.

Figure 6 shows the mapping robot. The controller of the robot is Jetson TX2, on which the Ubuntu 16.04 system and kinetic version of the Robot Operating System are installed. The laser scanning range finder installed above the robot is URG-04LX-UG01 2D laser scanning range finder produced manufactured by HOKUYO AUTOMATIC CO., LTD. The measuring distance is 5.6 m, measuring angle is 240°, and updating frequency is 10 Hz. The Kinect v1 RGB-D camera was installed above the laser sensor. The color resolution of the camera is 640 × 480 pixels; the resolution of the depth map is 320 × 240; moreover, the update frequency is 30 Hz. The horizontal and vertical fields of view are 57 and 43°, respectively. The chassis of the robot is fitted with a pair of differential drive wheels. The socketCAN node of the Robot Operating System is used to communicate with the motor driver, and the robot motion is controlled by a keyboard control node.

**Figure 7:**
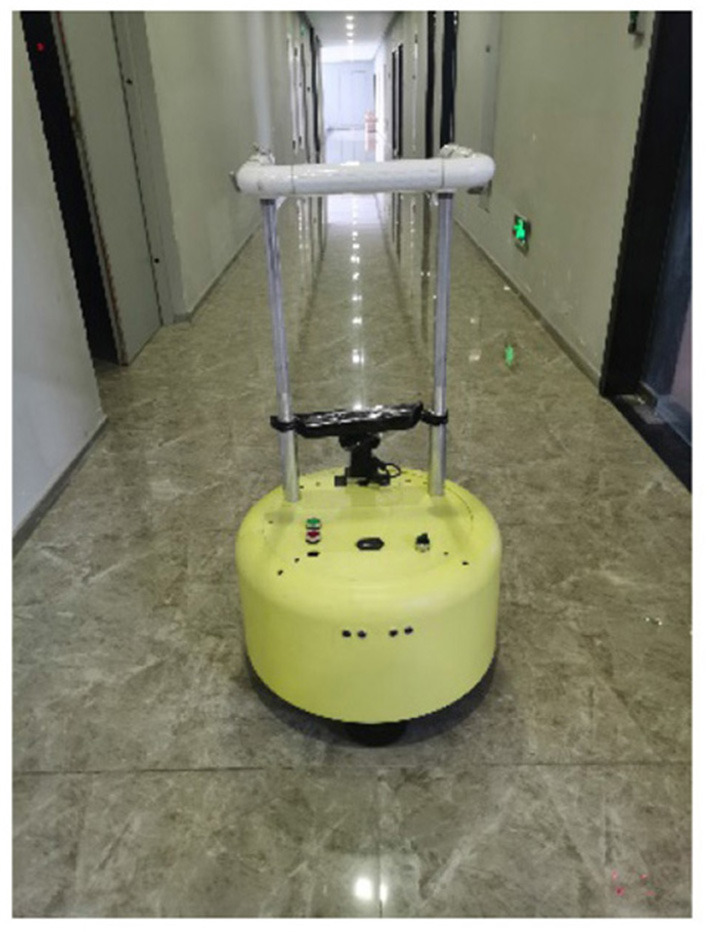
Real motion scene.

**Figure 8:**
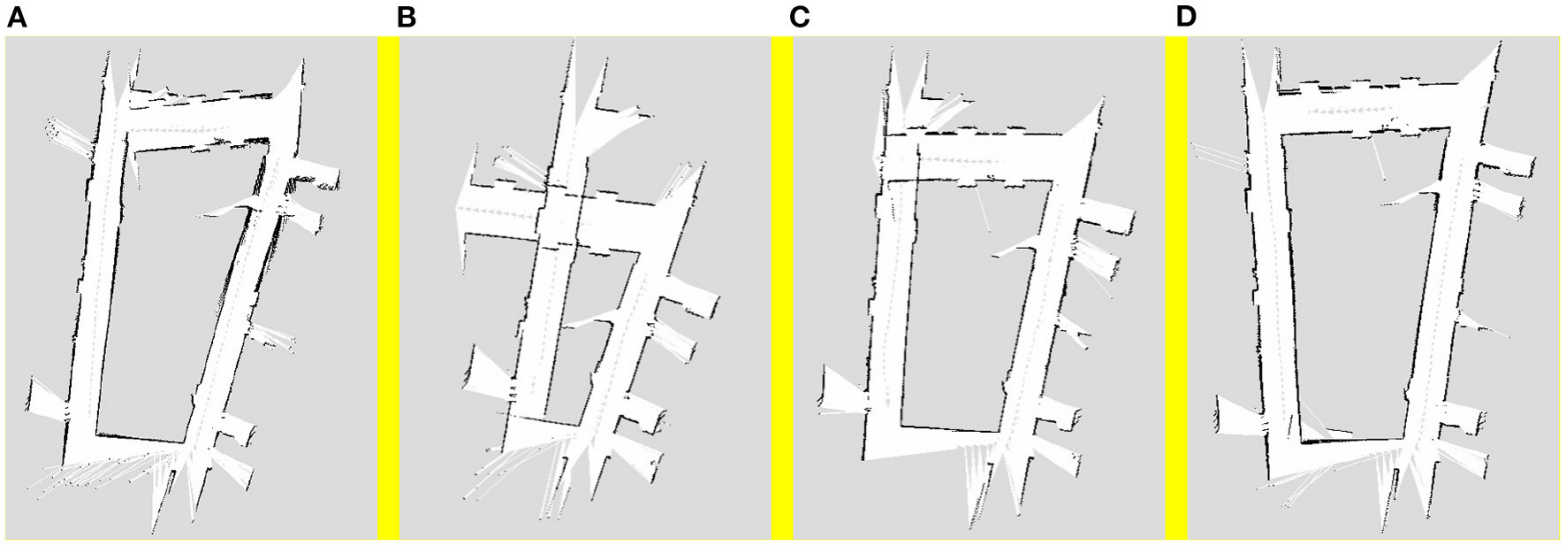
Mapping results of **(A)** pure visual algorithm; **(B)** pure laser algorithm; **(C)** Kalman filter algorithm; **(D)** weighted asynchronous fusion algorithm.

**Figure 9:**
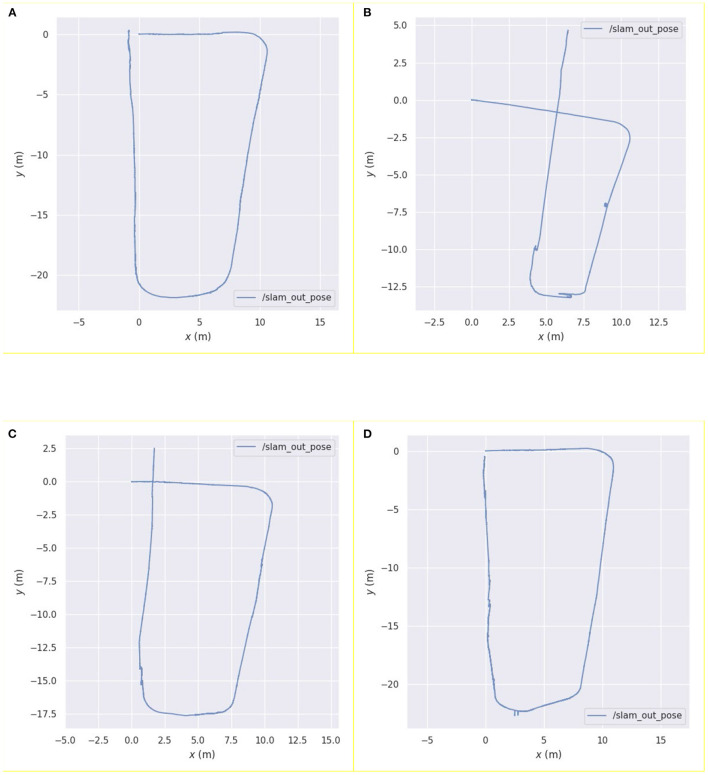
Robot trajectory results obtained using **(A)** pure vision algorithm; **(B)** pure laser algorithm; **(C)** Kalman filter algorithm; **(D)** asynchronous weighted fusion algorithm.

The scenes shown in Figure 7 are images of the real scene used for mapping, and the structure is a trapezoidal-shaped common first floor of an office building. A part of the middle area is a degraded scene where the robot moves and maps the area. The maps obtained using the four algorithms are shown in Figures 8A–D, respectively. The robot trajectory is shown in Figures 9A–D.

Figures 8A,B show the mapping results obtained using the visual and laser SLAM algorithms, respectively. Similar to the results in the simulation, the errors in pose results using vision data primarily occur in angular deviations, whereas the errors in pose results using the laser data predominantly occur during the pose estimation in the degraded environments. Figure 8C shows the mapping result obtained using the Kalman filter algorithm, which is obtained by combining the laser and visual pose estimations. This algorithm can reduce the influence of the degraded environment to a certain extent; however, there is still a large error. Figure 8D shows the trajectory results obtained using the weighted asynchronous Kalman filtering algorithm. When compared to the previous results, the result of this algorithm is the best among the four mapping results, which shows that the mapping accuracy of the weighted asynchronous Kalman filtering algorithm is better than the previous three algorithms.

Figure 9 shows the trajectory of the robot based on the pose estimations by the four algorithms. From the trajectory estimation results of the four algorithms, it can be observed that the pure laser algorithm has the largest estimation error in the degraded environment among the four algorithms, and the visual algorithm is prone to angular deviation. The starting and ending points in the trajectory estimation using the weighted asynchronous Kalman filter algorithm are basically the same, which shows that the positioning accuracy of the proposed algorithm is better than the previous ones.

## Conclusion

When compared to the traditional laser algorithm, the proposed weighted asynchronous multi-rate fusion algorithm can overcome the problem of inaccurate positioning in a degraded environment. The asynchronous fusion simultaneously achieves a higher tracking accuracy, fewer algorithm iterations, and shorter algorithm cycle times than ordinary fusion algorithms. The weighting algorithm can more effectively utilize the characteristics of different sensors and improve the positioning accuracy of the system in different scenarios. The current fusion mainly considers for pose estimation in the front-end, and we will consider adding it to loop detection to improve the accuracy of the algorithm in the future.

## Data Availability Statement

The raw data supporting the conclusions of this article will be made available by the authors, without undue reservation.

## Author Contributions

KX: conceptualization and funding acquisition. KX and YL: methodology. KX and XZ: software. KX, YL, XZ, WC, and WD: validation. YL and WD: formal analysis. XZ and WC: investigation. WD: data curation. KX, WC, and XZ: writing—original draft preparation. XZ and WC: writing—review and editing. WD: visualization and supervision. All authors have read and agreed to the published version of the manuscript.

## Funding

This work was supported by State Key Laboratory of Robotics and Systems (HIT) (SKLRS-2021-KF-11).

## Conflict of Interest

The authors declare that the research was conducted in the absence of any commercial or financial relationships that could be construed as a potential conflict of interest.

## Publisher's Note

All claims expressed in this article are solely those of the authors and do not necessarily represent those of their affiliated organizations, or those of the publisher, the editors and the reviewers. Any product that may be evaluated in this article, or claim that may be made by its manufacturer, is not guaranteed or endorsed by the publisher.
